# Collagen-Derived Peptides in CKD: A Link to Fibrosis

**DOI:** 10.3390/toxins14010010

**Published:** 2021-12-23

**Authors:** Emmanouil Mavrogeorgis, Harald Mischak, Agnieszka Latosinska, Antonia Vlahou, Joost P. Schanstra, Justyna Siwy, Vera Jankowski, Joachim Beige, Joachim Jankowski

**Affiliations:** 1 Mosaiques Diagnostics GmbH, 30659 Hannover, Germany; mavrogeorgis@mosaiques.de (E.M.); mischak@mosaiques-diagnostics.com (H.M.); latosinska@mosaiques.de (A.L.); siwy@mosaiques-diagnostics.com (J.S.); 2 Institute for Molecular Cardiovascular Research (IMCAR), RWTH Aachen University Hospital, 52074 Aachen, Germany; vjankowski@ukaachen.de; 3 Biotechnology Division, Biomedical Research Foundation of the Academy of Athens, 11527 Athens, Greece; vlahoua@bioacademy.gr; 4 Institut National de la Santé et de la Recherche Médicale (INSERM), U1297, Institute of Cardiovascular and Metabolic Disease, 31432 Toulouse, France; joost-peter.schanstra@inserm.fr; 5 Université Toulouse III Paul-Sabatier, 31062 Toulouse, France; 6 Division of Nephrology and KfH Renal Unit, Hospital St Georg, 04129 Leipzig, Germany; joachim.beige@kfh.de; 7 Department of Internal Medicine 2 (Nephrology, Rheumatology, Endocrinology), Martin-Luther-University Halle/Wittenberg, 06108 Halle/Saale, Germany; 8 Experimental Vascular Pathology, Cardiovascular Research Institute Maastricht (CARIM), University of Maastricht, 6229 Maastricht, The Netherlands

**Keywords:** CE-MS, chronic kidney disease, collagen alpha-1(I) chain, fibrosis, urine

## Abstract

Collagen is a major component of the extracellular matrix (ECM) and has an imminent role in fibrosis, in, among others, chronic kidney disease (CKD). Collagen alpha-1(I) (col1a1) is the most abundant collagen type and has previously been underlined for its contribution to the disease phenotype. Here, we examined 5000 urinary peptidomic datasets randomly selected from healthy participants or patients with CKD to identify urinary col1a1 fragments and study their abundance, position in the main protein, as well as their correlation with renal function. We identified 707 col1a1 peptides that differed in their amino acid sequence and/or post-translational modifications (hydroxyprolines). Well-correlated peptides with the same amino acid sequence, but a different number of hydroxyprolines, were combined into a final list of 503 peptides. These 503 col1a1 peptides covered 69% of the full col1a1 sequence. Sixty-three col1a1 peptides were significantly and highly positively associated (rho > +0.3) with the estimated glomerular filtration rate (eGFR), while only six peptides showed a significant and strong, negative association (rho < −0.3). A similar tendency was observed for col1a1 peptides associated with ageing, where the abundance of most col1a1 peptides decreased with increasing age. Collectively the results show a strong association between collagen peptides and loss of kidney function and suggest that fibrosis, potentially also of other organs, may be the main consequence of an attenuation of collagen degradation, and not increased synthesis.

## 1. Introduction

The collagen family consists of 28 different members and is the most abundant protein family in mammals [1,2]. About 30% of the human proteome is collagens, with this percentage being substantially higher in certain areas of the body (e.g., tendons) [3]. At least 90% of total human collagen comprises collagen type I [4]. Collagen type I is present in almost all connective tissues. It is the interstitial matrix collagen, organized in fibrils, that plays an important role in the structure of different tissues. Several post-translational modifications (PTMs) that underlie collagen type I, among which, hydroxylation of proline and lysine, as well as the glycosylation of the hydroxylated latter, occur during translation [1].

Collagen alpha-1(I) (col1a1), a type I fibrillary collagen protein, is the most abundant member of the collagen family and a major component of the extracellular matrix (ECM). Excessive accumulation of col1a1 is a key element of fibrosis [5]. Fibrosis refers to the extravagant accumulation of connective tissue in the ECM of an organ potentially leading to malfunction [6]. ECM consists of a basement membrane and an interstitial matrix, both of which contain a collagen scaffold with adhesive glycoproteins and proteoglycans interacting with cells within or near the matrix [7]. Noteworthy is the dynamic character of the ECM, which is continually remodeled in general through the enzymatic activity of proteases, usually matrix metalloproteinases, a disintegrin and metalloproteinases (ADAMs), ADAMs with thrombospondin motifs (ADAMTS), plasminogen activators, cathepsins and granzymes [8].

In several recent studies, a reduction of urinary col1a1 fragments was found to be associated with fibrosis-related pathologies. For example, in a fibrosis-focusing study, two specific col1a1 peptides, among other collagen fragments, demonstrated a negative correlation with the degree of fibrosis [9]. This is in agreement with the observation that the downregulation of the col1a1 degradation process appears to be associated with progression of fibrosis in heart failure [10]. In the context of obesity-related nephropathy, col1a1 fragments were also among the most significant peptides correlating (negatively) to body mass index (BMI) and (positively) to estimated glomerular filtration rate (eGFR) [11]. In another study, peptides from col1a1 were among the most promising ones to (positively) correlate with eGFR in both mild-moderate and advanced CKD [12].

Since the development and dynamics of the disease phenotype are directly attributed to the abundance and functionality of proteins, numerous publications rely on clinical proteomics [13] and peptidomics [14]. That said, a notable part of the CKD literature focuses on peptides analyzed through capillary electrophoresis coupled to mass spectrometry (CE-MS). The added value of CE-MS has been extensively described in the literature [15,16,17,18], while the focus on urine is based on its proximity to the kidneys. Through these studies, the association of collagens to CKD, notably col1a, was highlighted.

Building on these existing data, we aimed to decipher and emphasize the pivotal role of col1a1 fragments with regard to the pathophysiology of CKD. A secondary goal was to investigate on the impact of hydroxyprolines in the distribution of the col1a1 peptides. We used 5000 peptidomics datasets from the Human Urinary Proteome Database [16], obtained using CE-MS for the analysis of samples from healthy controls and patients with CKD. Investigated were 707 different col1a1 protein fragments detected in urine as well as their correlation with kidney function loss to annotate the contribution of col1a1 to CKD.

## 2. Results

### 2.1. Patient Characteristics

Urinary peptidomics data based on CE-MS technology were extracted from the Human Urinary Proteome Database [16]. We randomly extracted 5000 datasets from adult subjects, limiting to datasets from controls or patients with CKD, in which the estimated glomerular filtration rate (eGFR) and age were available. The underlying different CKD aetiologies are listed in Table 1.

The mean age of the participants was 55.79 (±15.82) years, ranging from 18.00 to 94.54 years. The eGFR of the participants ranged from 15.00 to 150.00 mL/min/1.73 m^2^, with a mean of 81.03 mL/min/1.73 m^2^. In detail, 2088 participants had eGFR ≥ 90 mL/min/1.73 m^2^, and 1253 participants had an eGFR < 60 mL/min/1.73 m^2^. For the remaining 1659 participants, the eGFR was in the interval of 60–90 mL/min/1.73 m^2^. Of note, a number of CKD patients demonstrated eGFR ≥ 90, but were diagnosed with CKD stage 1 due to albuminuria or other urine abnormalities indicative of CKD.

### 2.2. Identification of Unique col1a1 Protein Fragments

In the extracted datasets from the Human Urinary Proteome Database, we could detect 707 col1a1 peptides that were present in a minimum of 100 participants each. Of these, 359 peptides showed unique amino acid sequences. The remaining 348 peptides corresponded to 144 unique amino acid sequences with different numbers of PTMs (hydroxylation of proline).

**Figure 1:**
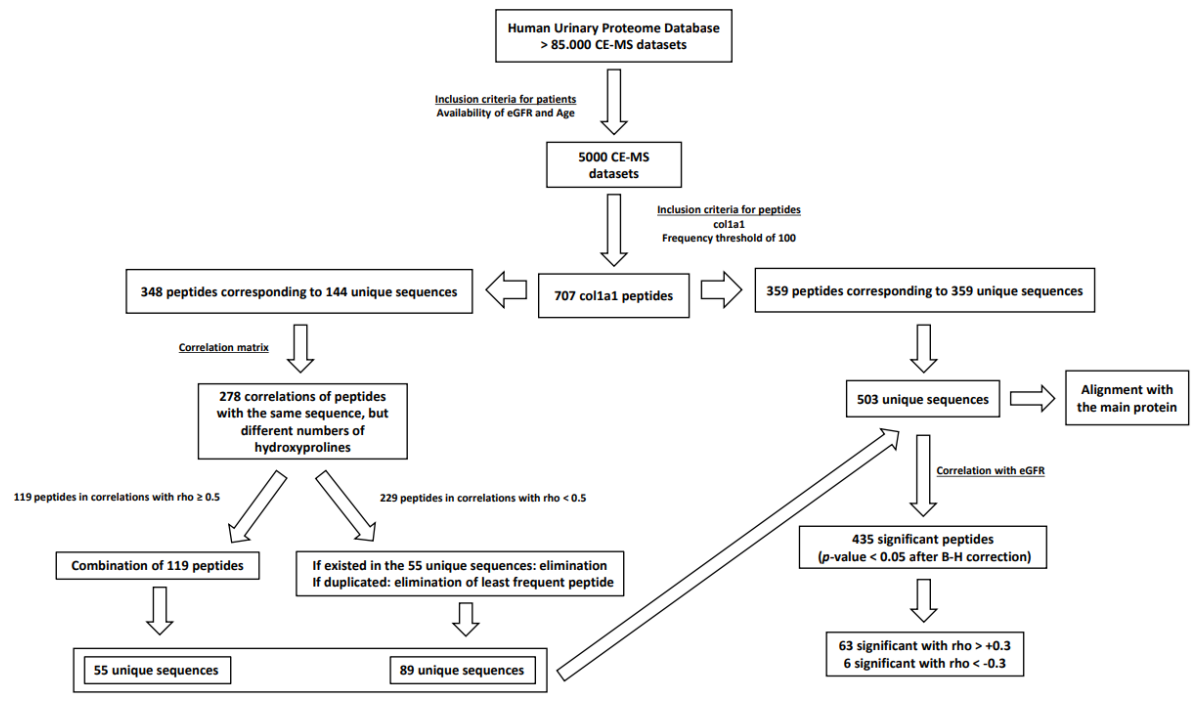
Study design. Datasets of 5000 participants were initially obtained from the Human Urinary Proteome Database. In this database, 707 col1a1 protein fragments that satisfied a frequency threshold of 100 participants, were detected. Of these, 359 peptides corresponded to unique amino acid sequences, while 348 peptides corresponded to 144 unique amino acid sequences, but included different numbers of PTMs. The correlation analysis of the latter (348 peptides) resulted in the identification of 119 peptides which were further combined due to their high rho values to 55 unique col1a1 fragments as well as 229 peptides that were not combined due to their low rho values, but were reduced to 89 unique col1a1 fragments. Thus, a final list of 503 unique col1a1 fragments was included in further analysis, which involved correlation with eGFR and also age. The results indicate that 435 peptides were significantly correlated with eGFR and 408 with age (*p*-values were corrected for multiple testing). All 503 sequences were used as an input for the alignment with the sequence of the main protein.

The study design is shown in Figure 1. As a first step, to reduce data complexity, the relation between identical protein fragments (identical amino acid sequence) with different numbers of PTMs (hydroxyprolines) was investigated. We explored the hypothesis that differences in proline hydroxylation should not show a significant impact on the peptide relative abundance, by assessing the correlation between peptides with identical sequence, but different proline hydroxylation. A rho value of at least 0.5 was considered as denoting association. Of the 348 peptides, 119 fulfilled this criterion. These 119 peptides corresponded to 55 unique sequences, which were then used in further analysis. The remaining 229 peptides, corresponding to 89 unique sequences, correlated with rho < 0.5. The lower rho value indicated that the underlying biological processes, resulting in the generation of the peptides, may be affected by the PTMs, and consequently, these data were not combined. After (a) removing from the 229 peptides those that corresponded to sequences already included in the list of 55 unique sequences and (b) keeping the most frequent peptides in case of duplicates, the 229 peptides were reduced to 89 peptides that corresponded to 89 unique amino acid sequences. These 89 unique amino acid sequences were then merged with the 55 protein fragments as well as the 359 peptides that corresponded to 359 unique amino acid sequences, resulting in total in 503 unique col1a1 peptides that were included in further analysis.

**Figure 2:**
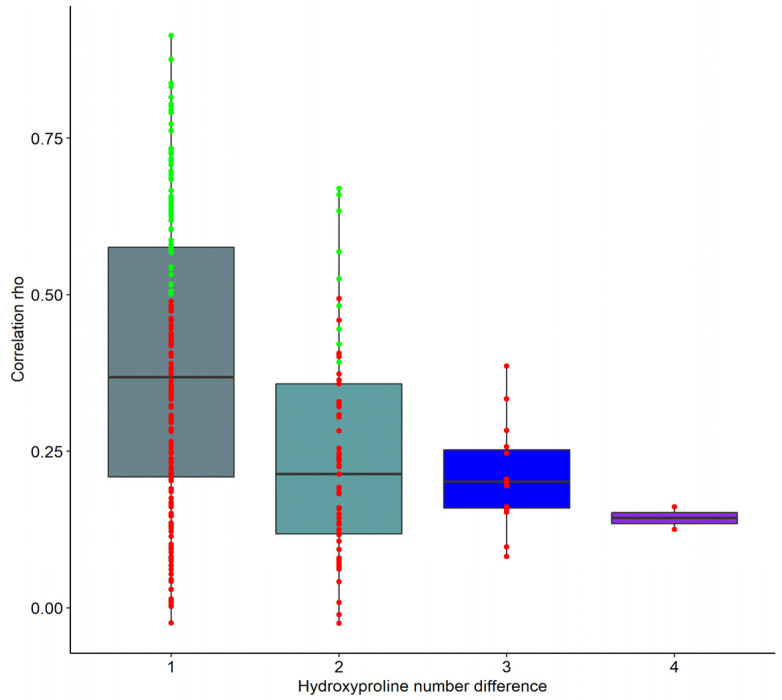
Boxplots of the Spearman’s rank correlation coefficient (rho) per hydroxyproline number difference. The graph illustrates a potential association between peptides with identical amino acid sequence, but differing in the number of hydroxyproline residues they carry (differences of *n* = 1, 2, 3 and 4). The green- and red-colored points represent the peptides that were (green) or were not (red) combined for the further analyses, based on the 0.5 rho threshold.

Since the number of peptides with an identical sequence that only differed in PTMs with rho < 0.5 was higher than expected (based on the initial hypothesis), we investigated the impact of the number of proline hydroxylation on the peptide correlation. In 278 pairs of peptides, the rho value was investigated concerning the difference in the number of hydroxyprolines. As evident from the data shown in Figure 2, the higher the difference in the number of hydroxyprolines was, the lower the correlated signal intensities of the respective protein fragments were. Supplementary Figure S1 provides an example of the correlation between identical (same amino acid sequence) protein fragments with different numbers of PTMs.

**Figure 3:**
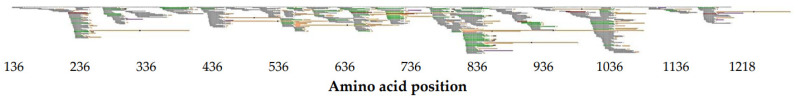
Alignment of 503 col1a1 fragments detected by CE-MS. Peptides were aligned to the col1a1 sequence from an amino acid position of 136 until 1218. The peptides correlated with eGFR (corrected for multiple testing) with rho > +0.3 are colored green, whereas the red color indicates rho < −0.3. The rest of the fragments are colored grey. The bars on the right of the sequences indicate the normalized relative total abundance of significantly (corrected for multiple testing) correlated (orange) or non-significantly correlated (purple) peptides with eGFR. On each bar, diamond, square or triangle black-colored shapes may appear in case the peptide belonged to the top ten: lowest *p*-values, highest total intensity or (absolute) Spearman’s rank correlation coefficient (rho), respectively. The figure is also provided online as Supplementary Figure S2 for more in-depth higher resolution studying.

The distribution of the 503 peptides concerning the col1a1 sequence is shown in Figure 3 as well as in high resolution in Supplementary Figure S2, which provides the opportunity to zoom in on the sequences. All peptides combined cover 69% of the entire amino acid sequence of col1a1. The lowest starting amino acid of the identified sequences aligns with the 136th amino acid of the main protein and likewise, their very last amino acid corresponds to the 1218th. Positions of the main protein for which no coverage was observed by the urinary col1a1 fragments are the following (in amino acid intervals): 1–135, 253–271, 577–578, 742–759, 954–959, 1075–1094, 1167–1168, 1219–1464 (C-terminus).

### 2.3. Correlation of Unique col1a1 Protein Fragments with eGFR and Age

In the next step, statistical analyses to define unique col1a1 fragments significantly associated with CKD as a prototype of a fibrotic disease were performed. As a measure for the disease stage, eGFR was used and correlation analysis of the signal intensities of the 503 protein fragments with the eGFR of the patients was performed (Supplementary Table S1). The correlation analysis revealed 435 peptides significantly associated (*p*-value < 0.05 after correction for multiple testing) with kidney function. The ten peptides that showed the strongest correlations (based on both rho and *p*-values) with eGFR demonstrated only positive correlations and are listed in Table 2.

To graphically depict the association of urinary col1a1 peptides with kidney function, the peptides aligned with the col1a1 sequence in Figure 3 were labelled as follows. Peptides displaying a strong positive correlation with eGFR (rho > +0.3) were marked in green. The few peptides demonstrating a strong negative correlation (rho < −0.3) were marked in red. Peptides moderately associated with eGFR were colored grey. Out of 503 col1a1 fragments, 63 were associated with eGFR with a rho > +0.3, while 6 demonstrated rho < −0.3.

An apparent “hotspot” in col1a1 peptides was observed between amino acids 765 and 854. This region segregates numerous sequences positively correlated with eGFR, eight of which are also among the top ten peptides with the highest correlation rho and lowest correlation *p*-value.

In contrast, the most abundant peptides do not appear to be concentrated in a specific area, and also generally do not display a strong association with eGFR and can be found dispersed across the col1a1 sequence. In detail, four showed practically no correlation with eGFR, two showed a negative correlation (rho = −0.159 and rho = −0.146) and four showed a positive correlation with rho values ranging from +0.171 to +0.338 (Supplementary Table S2).

As fibrosis and kidney function are associated with age–an association of several collagen fragments with age has been reported in [19,20,21]—we also investigated the association of the peptides with age, in the 5000 participants used in this study. Of the 503 peptides investigated, 408 revealed a significant association with age, 133 directly correlated to age and 275 inversely correlated (Supplementary Table S1). Of the ten most significant age-associated col1a1 peptides, six were also among the ten most significantly associated with kidney function. Age and kidney function are also generally correlated. To account for this fact, we first corrected eGFR for age, based on assuming a loss of 1 mL/min/1.73 m^2^ GFR per year, starting at the age of 30. As shown in Supplementary Table S1, the observed association of age and eGFR is lost upon correction for age, as expected. When investigating the correlation of the col1a1 peptide abundance with the age-corrected eGFR, the data obtained on the uncorrected eGFR are generally reproduced, although in most cases rho values are reduced. This may indicate that both age and eGFR, independent from each other, have an impact on the appearance of the urinary col1a1 fragments.

As it does not appear to be possible to directly adjust age for eGFR, we divided the study cohort into sub-cohorts of 10-year intervals (<30, 30–40, 40–50, 50–60, 60–70, >70 years) and then matched these six groups for eGFR. A total of 960 datasets (160 per age group) were defined in this way. In this dataset, no association of age with eGFR could be observed, as expected. Using these sub-cohorts, the association of the col1a1 peptides with age independent of eGFR was investigated. The results demonstrated a significant association of 244 peptides with age (91 peptides directly and 153 inversely correlated). The ten most significant and highly correlated peptides each associated with either eGFR corrected for age or with age in the cohort matched for eGFR, are listed in Table 3.

## 3. Discussion

To the best of our knowledge, this is the first comprehensive report on urinary col1a1 derived peptides at high resolution, and in a large cohort of 5000 subjects. Collagen alpha-1 (I) is the most abundant protein in the human body, its homeostasis is of the utmost importance and is a result of synthesis and degradation. While synthesis, assessed via gene expression, has been investigated in large detail, far less is known about its degradation [22,23].

The structure of collagen is well described [24]. Collagen is a structural motif formed when three parallel polypeptide strands construct a triple helix. This requires that every third residue in the helical conformation is glycine, although this pattern is disrupted at specific points in non-fibrillary collagens. On this pattern, the first and second amino acids are usually (2S)-proline (28%) and (2S,4R)-4-hydroxyproline (38%), respectively. The triple-helical conformation is responsible for the complexity and hierarchy of fibers and networks that collagens participate in. That said, hydroxyprolines occupying the second amino acid position of the pattern significantly enhance the thermal stability of the triple helix [25,26], as long as they do not occupy at the same time the first amino acid position of the pattern or the hydroxyl group is not in the 4S form as in (2S,4S)-4-hydroxyproline [27,28].

Collagen is unique among proteins as it contains abundant amounts of hydroxylated proline. In general, this PTM is added at the time of assembly into triple helices, almost simultaneously with translation. Collagen peptides also represent the by far most abundant group of peptides in human urine. This may to some degree also be the result of hydroxyproline. This amino acid cannot be re-used for protein synthesis and, as such, may be a signal to exclude the respective peptide from tubular reabsorption, further enriching for hydroxyproline containing peptides in urine as suggested by He et al. [29].

An age-related modification reported in collagen [30] is glycation, occurring non-enzymatically between, among others, proteins and reducing sugars [31]. Since a slow protein turnover rate favors the accumulation of advanced glycation end-products (AGEs) [32] and the rate of collagen turnover in humans is low, in some studies reported to be at least 10 years [30,32], structural and functional collagen properties may be impacted in such accumulation. That said, AGEs have been reported [30] as key modifications in collagen: by affecting the intermolecular crosslinking of neighboring fibers [33] and influencing the collagen interaction sites [34] (e.g., used for interactions with metalloproteinases) collagen viscoelasticity is decreased [35], while at the same time, the potential of degradation and replacement by newly synthesized molecules is diminished as the reported association between AGEs and reduced metalloproteinase expression indicates [36,37], along with the enhanced resistance to proteolytic enzymes that AGEs provide to collagens [38]. The collagen cross-linking mediated non-enzymatically by AGEs occurring as Maillard reactions between lysine and histidine or arginine residues [39], differs from the enzymatically-produced cross-linking, that occurs due to covalent bonds formed between the C- and N-termini of neighboring lysine or hydroxylysine groups, hence the altered biomechanical properties of glycated collagen molecules [40].

Accumulation of collagen is a hallmark in fibrosis in general, including fibrosis in the context of CKD. While the synthesis of collagen has been investigated in great detail, based on gene expression, the biological processes responsible for its degradation are far less well described. However, as also outlined in a recent review, the abundance of collagen is the result of both, synthesis and degradation [41]. To assure homeostasis, which is essential to preserve the structure of the body and organs, both, synthesis and degradation have to be in balance. With this manuscript, we aimed to shed some more light on col1a1 degradation under physiological conditions, via assessing the degradation products, the collagen peptides. The study was also driven by the hypothesis that col1a1 degradation is attenuated in fibrosis, using CKD as a prototypic example for fibrotic disease.

To this end, 707 col1a1 fragments were initially identified by CE-MS corresponding to 503 unique amino acid sequences after adjusting for the different hydroxyproline PTMs.

As expected, based on previous reports, most of the peptides were positively correlated with eGFR. When investigating the peptides strongly correlating (absolute rho value > +0.3) with eGFR, the ratio of positive vs. negatively associated peptides was 10.5:1. The few exceptions of negative peptide correlation with eGFR may be directly attributed to the high abundance of overlapping peptides with opposite correlation to eGFR.

When investigating the distribution of the few highly negatively (rho < −0.3) correlated significant peptides, it appears that the first and last peptides, that show such an inverse association with eGFR, originate from the N- and C-termini of the mature col1a1, respectively. This observation is similar to an observation recently reported by He et al. [10], where the authors found an increase of N- and C-terminal collagen type I fragments associated with death in the context of heart failure, while the peptides from the central part of collagen type I that were significantly associated with death in this prospective study were all reduced. Based on these data, the hypothesis was presented that increasing cross-linking over time as a result of chemical modification due to ageing, inflammation or diabetes renders collagen fibers more resistant to degradation. This process is more prominent in the central part of the molecule. Increased resistance to degradation may result in an increase of protease activity, which however only leads to increased degradation of the collagen termini, while the central part of the molecule is largely protected due to the acquired crosslinking.

In a recent review [22], the association between urinary collagen peptides and various CKD aetiologies was described. In these studies, especially col1a1 peptides were described as positively correlated with both mild-moderate and advanced CKD [12] as well as negatively correlated with fibrosis [9] and obesity-related nephropathy [11]. In a recent paper focusing on a 29-peptide classifier (five of which were col1a1 peptides) to evaluate interstitial fibrosis and renal atrophy in a non-invasive approach [42], three urinary col1a1 peptides were reported to negatively correlate with interstitial fibrosis and renal atrophy. Multiple col1a1 fragments have also been described associated with aging and age-related chronic diseases [19].

The presence of different CKD aetiologies is expected to have a different impact on the molecular pathophysiology of the disease and as such, in the proposed urinary collagen peptides. To investigate this topic, an additional study on a large cohort that is also balanced for the different CKD aetiologies is warranted in order to decipher the aetiology-related impact on fibrosis and the correspondent urinary collagen profiles.

The CE-MS technique utilized in our paper led to the identification of peptide fragments that cover 69% of the col1a1 protein sequence. However, the full col1a1 sequence also contains the “Signal peptide” (1–22), “N-terminal propeptide” (23–161) and the “C-terminal propeptide” (1219–1464), all of which are cleaved upon generation of the mature collagen protein, which encompasses amino acids 162–1218. As evident from the data, almost no peptide derived from sequence outside the mature col1a1 protein was detected in the urine in this study. This further suggests that the peptides identified do reflect degradation of the mature collagen and are not connected to any process in collagen synthesis and assembly of the mature protein. That said, peptides identified in this study covered almost 94% of the mature col1a1 protein.

Looking to the future, the selected specific urinary col1a1 fragments defined here can be integrated in a multi-dimensional classifier that could be implemented into clinical practice. Urinary peptide-based classifiers have been described in the past, such as the CKD273 [43] for chronic kidney disease or the recent COV50 [44] for SARS-CoV-2-infected patients. Moreover, a classifier based on 29 urinary peptides originating from different proteins has been recently described as able to predict the degree of renal interstitial fibrosis and tubular atrophy (IFTA) in CKD patients [42]. The generation of a classifier based on here defined col1a1 peptides requires validation in an independent set of samples. We expect that the technology used for the analysis of the selected col1a1 peptides will be based on mass spectrometry. Immunological techniques are frequently used in routine analysis (e.g., ELISA), but they appear to be of insufficient selectivity to address specific peptides.

## 4. Conclusions

In this study, we provide a detailed and comprehensive map and description of urinary collagen alpha-1(I) fragments and their changes in the context of CKD and ageing. The study suggests that collagen degradation is attenuated in both, kidney disease and ageing, implying that fibrosis in humans may be the consequence of impaired collagen degradation and possibly to a lesser degree due to an increase in collagen synthesis. The results presented are expected to lay the foundation for the non-invasive assessment of fibrosis in the kidney, but also in other organs, based on specific urinary collagen peptides.

## 5. Materials and Methods

### 5.1. Patients

Patient data were acquired from the Human Urinary Proteome database, which contains urinary peptides datasets analyzed through CE-MS. This database includes more than 85,000 datasets processed and normalized as described before [16,45,46]. This approach results in highly comparable datasets, with no detectable batch effects [18,47].

Our inclusion criterion was participants with available eGFR and age information, either healthy volunteers or CKD patients. With this approach, randomly selected 5000 participants were considered for the analysis. Participants belonged to studies already described in the literature [12,42,48,49,50,51]. The underlying disease aetiologies can be found in Table 1. The study is in agreement with the Declaration of Helsinki [52], all participants gave written participation consent and the data collected were anonymized.

### 5.2. Data Curation

The estimation of GFR was based on the Chronic Kidney Disease Epidemiology Collaboration (CKD-EPI) equation [53].

### 5.3. Statistical Analysis

A threshold of detection in a minimum of 100 datasets was applied in the identified sequenced peptides as a requirement to be considered for further analyses. The correlation analysis was based on Spearman’s rank method. Correction for multiple testing was based on the Benjamini and Hochberg method and the threshold of significance was an adjusted *p*-value < 0.05. For the aggregation of the intensities of identical protein fragments with different numbers of hydroxyprolines into one protein fragment, the rho threshold of 0.5 was used.

The results and findings of the current paper were based on R programming (R version 4.1.0, R Foundation for Statistical Computing, Vienna, Austria). The correlation matrix was created with the rcorr function of Hmisc R package. The correlation of the peptides with eGFR was performed with the function cor.test of the stats R package. The col1a1 sequence was retrieved from Uniprot via the getUniProt function of the protr package [54]. The FASTA files required for the alignment procedure were created with the function dataframe2fas of seqRFLP package. The alignment of the peptides to the col1a1 sequence was based on the R package ggmsa. Matching between age sub-cohorts based on the eGFR values at 1:1 ratio was performed in R (‘MatchIt’) using ‘nearest neighbor’ method [55]. Distance-measure was estimated with logistic regression. The plots were based on the package ggplot2 [56]. The plots in Supplementary Figure S1 were arranged in the same figure using ggpubr R package.

## Figures and Tables

**Table 1:** Number of samples per disease aetiology investigated in the study.

Disease Aetiology	Number
Controls	1717
Amyloidosis	3
Diabetes mellitus	2756
Focal segmental glomerulosclerosis	27
IgA nephropathy	247
Minimal change disease	16
Membranous glomerulopathy	28
Membranoproliferative glomerulopathy	2
Nephritis	3
Nephrosclerosis	135
Systemic lupus erythematosus	22
Steroid-Resistant Nephrotic Syndrome	4
Vasculitis	40

**Table 2:** Top ten strongest correlations with eGFR based on the (absolute) Spearman’s rank correlation coefficient (rho) and *p*-values. The association of these peptides with age is also listed. AA: amino acid. p: Hydroxyproline.

Sequence	Start AA	Stop AA	rho eGFR	eGFR *p*-Value (B-H)	rho Age	Age *p*-Value (B-H)
ADGQpGAKGEpGDAGAKGDAGpPGPAGPAGPpGPIG	819	854	0.61	0.00	−0.39	1.14 × 10^−180^
IGPpGPAGApGDKGESGPSGPAGPTG	769	794	0.59	0.00	−0.38	7.36 × 10^−172^
LTGPIGppGPAGAPGDKGESGPSGPAGPTG	765	794	0.57	0.00	−0.36	1.19 × 10^−153^
pPGADGQPGAKGEpGDAGAKGDAGppGPAGPAGPPGPIG	816	854	0.55	0.00	−0.34	2.69 × 10^−132^
PpGPAGFAGPPGADGQPGAKGEpGDAGAKGDAGPPGPAGP	807	846	0.54	0.00	−0.31	7.21 × 10^−110^
LDGAKGDAGPAGPKGEpGSpGENGApG	273	299	0.50	0.00	−0.38	5.05 × 10^−169^
TGPIGpPGPAGAPGDKGESGpSGPAGPTG	766	794	0.50	0.00	−0.29	3.64 × 10^−94^
GPpGADGQPGAKGEpGDAGAKGDAGPPGpAGPAGPPGpIG	815	854	0.50	1.02 × 10^−305^	−0.35	3.06 × 10^−142^
GADGQpGAKGEpGDAGAKGDAGPPGPAGPAGPpGPIG	818	854	0.48	1.42 × 10^−291^	−0.27	6.08 × 10^−81^
NGDDGEAGKPGRpGERGPpGPQG	229	251	0.48	3.41 × 10^−279^	−0.25	3.26 × 10^−69^

**Table 3:** Top ten strongest (based on absolute Spearman’s rank correlation coefficient (rho) and *p*-values) correlations with age-corrected eGFR (top) or with age in eGFR-matched subjects (bottom). Each set of ten peptides is ordered based on the respective bold, B-H corrected *p*-value. The two peptides that are among the ten most significant in both comparisons are indicated in red. The generally lower *p*-value for association with age is a result of a lower number of subjects in this dataset (960 vs. 5000) and should not be interpreted as generally lower significance in comparison to kidney function. AA: amino acid. p: Hydroxyproline. m: Methionine sulfoxide.

**Sequence**	**Start AA**	**Stop AA**	**rho** **Age-corrected eGFR**	**Age-corrected eGFR** ***p*-value** **(B-H)**	**rho** **Age-matched**	**Age-matched *p*-value** **(B-H)**
ADGQpGAKGEpGDAGAKGDAGpPGPAGPAGPpGPIG	819	854	0.44	**2.84** **×** **10** ^−**232**^	−0.27	4.28 × 10^−16^
PpGPAGFAGPPGADGQPGAKGEpGDAGAKGDAGPPGPAGP	807	846	0.41	**1.64** **×** **10** ^−**202**^	−0.15	1.92 × 10^−05^
IGPpGPAGApGDKGESGPSGPAGPTG	769	794	0.41	**1.05** ** × ** **10^−201^**	−0.28	4.70 × 10 ^−17^
PGPAGPPGEAGKPGEQGVPGDLGAPGPSGARG	646	677	−0.41	**9.43** **×** **10** ^−**197**^	−0.06	1.47 × 10^−01^
LTGPIGppGPAGAPGDKGESGPSGPAGPTG	765	794	0.40	**1.24** **×** **10** ^−**191**^	−0.27	5.95 × 10^−16^
pPGADGQPGAKGEpGDAGAKGDAGppGPAGPAGPPGPIG	816	854	0.40	**4.80** ** × ** **10** ^−**186**^	−0.29	6.76 × 10 ^−19^
TGPIGpPGPAGAPGDKGESGpSGPAGPTG	766	794	0.38	**1.26** **×** **10** ^−**168**^	−0.24	1.22 × 10^−12^
NGDDGEAGKPGRpGERGPpGPQG	229	251	0.37	**4.25** **×** **10** ^−**163**^	−0.22	1.15 × 10^−10^
KEGGKGPRGETGPAGRpGEVGPpGPpGPAG	903	932	0.37	**8.82** **×** **10** ^−**160**^	−0.10	6.96 × 10^−03^
GADGQpGAKGEpGDAGAKGDAGPPGPAGPAGPpGPIG	818	854	0.37	**1.68** **×** **10** ^−**158**^	−0.21	6.90 × 10^−10^
**Sequence**	**Start AA**	**Stop AA**	**rho** **Age-corrected eGFR**	**Age-corrected eGFR** ***p*-value** **(B-H)**	**rho** **Age-matched**	**Age-matched *p*-value** **(B-H)**
DAGPAGPKGEpGSpGENGApG	279	299	0.21	2.87 × 10^−52^	−0.39	**2.15** **×** **10^−33^**
LDGAKGDAGPAGPKGEpGSpGENGApG	273	299	0.31	9.95 × 10^−111^	−0.36	**5.16** **×** **10^−29^**
pGpAGEKGSpGADGPAGAP	928	946	0.02	2.58 × 10^−01^	0.36	**1.10** **×** **10^−28^**
GLPGpAGppGEAGKPGEQGVPGDLGApGP	644	672	0.16	2.92 × 10^−30^	−0.36	**1.68** **×** **10^−28^**
ADGQpGAKGEpGDAGAKGDAGPPGPAGP	819	846	0.30	1.83 × 10^−101^	−0.35	**5.81** **×** **10^−27^**
GSpGSpGPDGKTGPpGPAG	542	560	0.21	4.83 × 10^−48^	−0.35	**2.43** **×** **10^−26^**
EpGSpGENGAPGQmGPR	288	304	0.09	3.28 × 10^−11^	0.32	**3.01** **×** **10^−22^**
pPGADGQPGAKGEpGDAGAKGDAGppGPAGPAGPPGPIG	816	854	0.40	4.80 × 10 ^−186^	−0.29	**6.76** ** × ** **10^−19^**
EGSPGRDGSPGAK	1021	1033	0.15	4.99 × 10^−26^	0.28	**2.41** **×** **10^−17^**
IGPpGPAGApGDKGESGPSGPAGPTG	769	794	0.41	1.05 × 10 ^−201^	−0.28	**4.70** ** × ** **10^−17^**

## Data Availability

Data will be made available upon request directed to the corresponding author. Proposals will be reviewed and approved by the investigators and collaborators based on scientific merit. After approval of a proposal, data will be shared through a secure online platform after signing the data access and confidentiality agreement.

## Supplementary Materials

The following supporting information can be downloaded at: https://www.mdpi.com/article/10.3390/toxins14010010/s1, Figure S1: Correlation example of identical sequences with different PTMs, Figure S2: Col1a1 alignment plot, Table S1: Correlations of 503 unique col1a1 fragments identified by CE-MS, Table S2: Top ten most abundant col1a1 fragments identified by CE-MS.
