# Supplementary figures and images for: Collagen-Derived Peptides in CKD: A Link to Fibrosis
Source: Toxins (Basel). 2021 Dec 23;14(1):10. doi: 10.3390/toxins14010010 (PMC8781252; doi:10.3390/toxins14010010)

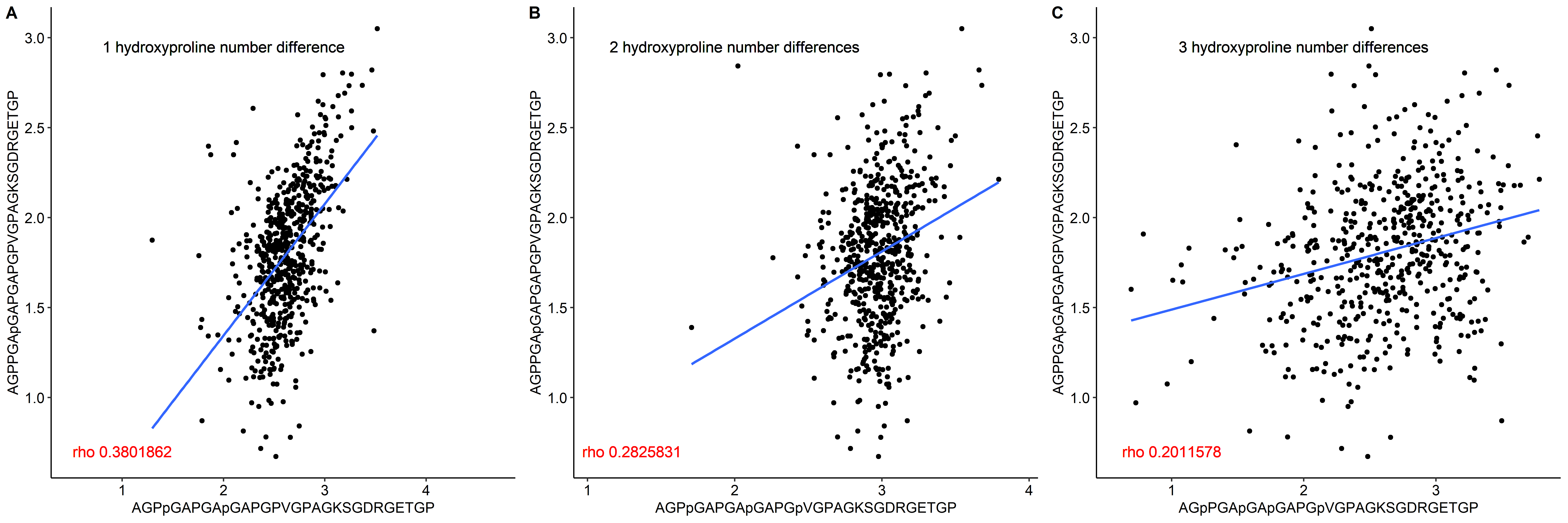

Supplement: Supplementary file 1 [file toxins-14-00010-s001.zip › Supplementary Figure S1 - Correlation example of identical sequences with different PTMs.png]
